# Effects of combined ciprofloxacin and Neulasta therapy on intestinal pathology and gut microbiota after high-dose irradiation in mice

**DOI:** 10.3389/fpubh.2024.1365161

**Published:** 2024-05-14

**Authors:** Timothy S. Horseman, Andrew M. Frank, Georgetta Cannon, Min Zhai, Matthew G. Olson, Bin Lin, Xianghong Li, Lisa Hull, Mang Xiao, Juliann G. Kiang, David M. Burmeister

**Affiliations:** ^1^School of Medicine, Uniformed Services University of the Health Sciences, Bethesda, MD, United States; ^2^Armed Forces Radiobiology Research Institute, Uniformed Services University of the Health Sciences, Bethesda, MD, United States

**Keywords:** gut microbiota, gastrointestinal, acute radiation syndrome, Neulasta, ciprofloxacin

## Abstract

**Introduction:**

Treatments that currently exist in the strategic national stockpile for acute radiation syndrome (ARS) focus on the hematopoietic subsyndrome, with no treatments on gastrointestinal (GI)-ARS. While the gut microbiota helps maintain host homeostasis by mediating GI epithelial and mucosal integrity, radiation exposure can alter gut commensal microbiota which may leave the host susceptible to opportunistic pathogens and serious sequelae such as sepsis. To mitigate the effects of hematopoietic ARS irradiation, currently approved treatments exist in the form of colony stimulating factors and antibiotics: however, there are few studies examining how these therapeutics affect GI-ARS and the gut microbiota. The aim of our study was to examine the longitudinal effects of Neulasta and/or ciprofloxacin treatment on the gut microbiota after exposure to 9.5 Gy ^60^Co gamma-radiation in mice.

**Methods:**

The gut microbiota of vehicle and drug-treated mice exposed to sham or gamma-radiation was characterized by shotgun sequencing with alpha diversity, beta diversity, and taxonomy analyzed on days 2, 4, 9, and 15 post-irradiation.

**Results:**

No significant alpha diversity differences were observed following radiation, while beta diversity shifts and taxonomic profiles revealed significant alterations in *Akkermansia*, *Bacteroides*, and *Lactobacillus*. Ciprofloxacin generally led to lower Shannon diversity and *Bacteroides* prevalence with increases in *Akkermansia* and *Lactobacillus* compared to vehicle treated and irradiated mice. While Neulasta increased Shannon diversity and by day 9 had more similar taxonomic profiles to sham than ciprofloxacin-or vehicle-treated irradiated animals. Combined therapy of Neulasta and ciprofloxacin induced a decrease in Shannon diversity and resulted in unique taxonomic profiles early post-irradiation, returning closer to vehicle-treated levels over time, but persistent increases in *Akkermansia* and *Bacteroides* compared to Neulasta alone.

**Discussion:**

This study provides a framework for the identification of microbial elements that may influence radiosensitivity, biodosimetry and the efficacy of potential therapeutics. Moreover, increased survival from H-ARS using these therapeutics may affect the symptoms and appearance of what may have been subclinical GI-ARS.

## Introduction

Pharmacological options for radiation exposures from nuclear or radiological events that lead to acute radiation syndrome (ARS) focus on hematopoietic symptoms. Higher doses of irradiation (> 6 Gy) induce gastrointestinal system subsyndrome (GI-ARS) resulting in diarrhea, vomiting, and potential mortality (1). The effects of GI-ARS are characterized by loss of crypts inhibiting intestinal cell proliferation and migration, apoptosis, villus blunting and fusion with mucosal epithelial barrier breakdown, electrolyte and nutrient imbalance, and inflammation (2). Radiation-induced GI injury is accompanied by bone marrow suppression and linked to systemic consequences such as multi-organ dysfunction (3, 4).

Irradiation compromises gastrointestinal barrier integrity (i.e., GI-ARS) increasing the risk of infectious complications such as sepsis (5, 6). Recently, commensal gut microbiota has been implicated as a critical component of host immunity and barrier integrity in health and a variety of disease states (7). Alterations or imbalances in microbial composition (termed dysbiosis) lead to inflammation and potential pathogen colonization (8–10). While leveraging the gut microbiota for biodosimetry and therapeutic targeting in ARS is of great interest, there are limited data examining the extent of dysbiosis following ionizing radiation.

As the radiation levels needed to induce GI-ARS are ethically difficult to explore in humans, existing studies using lower doses in radiotherapy can inform on potential changes seen in the microbiome. However, limitations include the impact that cancer itself has on the gut microbiota (11). In humans, GI-ARS leads to mortality 2–3 weeks post-radiation exposure (12). A mass casualty nuclear event would require a substantial public health effort including the use of the Strategic National Stockpile (SNS) that has been established to immediately distribute medical countermeasures (MCMs) and supplies. The SNS includes broad-spectrum antibiotics, medical items, and nuclear/radiological-specific resources such as chelating agents, Prussian blue, and growth factors/cytokines (13). Depending on total radiation doses, supportive care can range from antacids, antibiotics, hydration, and analgesics to the administration of blood cell transfusions if there is severe bone marrow damage (14). There are currently six FDA-approved drugs for the treatment of hematopoietic ARS (H-ARS) including PEGylated Granulocyte Colony Stimulating Factor (also known as PEG-G-CSF, Neulasta™, or PEG-filgrastim), which is in the SNS (15). Although these therapies can alleviate some H-ARS effects, a poly-pharmacy approach with an SNS resource such as ciprofloxacin (an FDA-approved antimicrobial) could enhance the efficacy of Neulasta which may improve outcomes that have been evident with inhibition of the radiation-induced brain hemorrhage in irradiated mice (16, 17). Small and large animal models are invaluable in the development of MCMs, testing of drug combinations, and understanding the mechanisms of radiation injury at higher doses (18). To this end, no FDA-approved MCMs exist for the treatment of GI-ARS and the impact of H-ARS therapies on GI symptoms induced by higher radiation doses is lacking (12).

Animal models have led to foundational knowledge on the effects of GI-ARS including gut microbiota responses (11). The literature suggests that radiation can lead to increases in opportunistic pathogens and decreases in commensal flora in the gut (19–23). Taxonomic classifications have been somewhat discordant between studies. However, generally an altered Firmicutes/Bacteroidetes ratio, *Lactobacillus* prevalence decreases, and increases in *Enterobacteriaceae* are observed post-irradiation (19–23). Although evidence suggests radiation alters the gut microbiota, not all studies are in agreement, and the lack of consonant results across studies underscores the importance of additional exploration of the gut microbiome post-radiation (19, 20, 23). Furthermore, the effect of drugs utilized for supportive care post-irradiation on the gut microbiota has not been elucidated to date.

To this end, the objective of this study was to evaluate the combined effect of ciprofloxacin and Neulasta on gut histopathology and microbiota in a mouse model of high-dose irradiation because this combined therapy has been shown to effectively mitigate radiation-induced brain hemorrhage (17). This model provides clinically relevant data on whether combined therapy reduces intestinal injury after irradiation. Additionally, we aim to uncover specific GI microbiota diversity and taxa alterations to provide diagnostic and therapeutic targets in response to radiation exposure. We hypothesized that Neulasta may positively protect gut flora from alterations caused by ciprofloxacin administration and radiation exposure (e.g., reduced alpha diversity), leading to microbiome restoration and alleviation of GI-ARS.

## Methods

### Mice

B6D2F1/J female mice (*n* = 192) from Jackson Laboratory (Bar Harbor, ME), 12 weeks old and approximately 20–26 g, were housed in an Association for Assessment and Accreditation of Laboratory Animal Care, International (AAALAC International) accredited facility. As previously described, male mice were excluded because of potential aggressive behavior (17). Mice were randomly assigned into different experimental groups with *n* = 6 per group per time point performed at two different time intervals with equal representation of each group within the study cohorts. There were both sham-irradiated (s) and irradiated (r) animals with four treatments in each cohort [vehicle (Veh); ciprofloxacin (CIP); PEGylated granulocyte colony-stimulating factor (Neulasta^®^, NEU); or combination (CIP + NEU)]. These mice were provided with commercial rodent chow (Rodent Diet #8604, Harlan Teklad, Madison, WI) and acidified tap water (pH = 2.5–2.8) *ad libitum*. Rooms holding animals were maintained at 22°C ± 2°C with 50% ± 20% relative humidity using at least 10–15 air changes/h of 100% conditioned fresh air with a 12-h, 0600 (light) to 1800 (dark), full-spectrum lighting cycle. Mouse tails were tattooed for individual identification during acclimation. All animal handling and experimentation were performed in accordance with an approved protocol by the Uniformed Services University of the Health Sciences Institutional Animal Care and Use Committee (IACUC).

### Gamma irradiation

On day 0, mice were restrained in a well-ventilated Lucite^®^ box and received 9.5 Gy ^60^Co γ-photon radiation (LD50/30; approximately 0.4 Gy/min) bilaterally at the Armed Forces Radiobiology Research Institute (AFRRI) high-level Co-60 facility (Nordion Inc., Ottawa, Canada). There were 24 mice irradiated simultaneously in the cohort; groups were equally represented. The alanine/electron spin resonance (ESR) dosimetry system (American Society for Testing and Material Standard E 1607) was used to measure dose rates to water in cores of acrylic mouse phantoms. The ESR signals were measured against a calibration curve based on standard calibration dosimeters provided by the National Institute of Standards and Technology (24). Mice were returned to their home cage post-irradiation for recovery and monitoring.

### Preparation and administration of ciprofloxacin and Neulasta

CIP [National Drug Code (NDC): 63739-700-10] was purchased from Aurobindo Pharma Limited (Hyderabad, India). For the 30-day survival study, CIP at 90 mg/kg was orally administered 2 h after irradiation and once daily up to 14 days thereafter. The vehicle given to sham mice was drinking water (17).

PEGylated G-CSF [Neulasta^®^ (NEU); NDC: 555-13-019001] is a polyethylene glycol pharmaceutical-formulated-grade drug, also known as pegfilgrastim, that was purchased from the AmerisourceBergen Corporation (Valley Forge, PA). NEU at a dose of 1,000 μg/kg was administered by s.c. injections (17) in a volume of 0.2 mL at 24 h, day 8 and day 14 after irradiation, i.e., 25 μg/25 g mouse. NEU was supplied in 0.6 mL prefilled syringes for s.c. injection. Each syringe contained 6 mg NEU in a sterile, clear, colorless, preservative-free solution containing 0.35 mg acetate, 0.02 mg polysorbate 20, 0.02 mg sodium, and 30 mg sorbitol in water for injection (United States Pharmacopeia). The vehicle mouse received 0.2 mL of a vehicle containing 0.35 mg of acetate, 0.02 mg of polysorbate 20, 0.02 mg of sodium, and 30 mg of sorbitol in 0.6 mL of water (17). CIP and NEU dosing regimens based on previously published studies (25, 26).

### Sample collection

Separate cohorts of mice were euthanized on days 2, 4, 9, and 15 in order to collect blood, tissues, and fecal pellets post-treatment/post-irradiation for a total of 4 time points (*n* = 6/group per time point). Mice were anesthetized by isoflurane inhalation at a metered range of 3–5% mixed with 100% oxygen gauged at 500–1000 cc/min in the isoflurane chamber. Then, the anesthetized mice were moved into a biological safety cabinet, with their noses placed in a funnel that was connected to the isoflurane instrument, and blood was collected through a cardiac punch. The cervical dislocation was performed to confirm death after blood collection. Then, a section of ileum was harvested and placed into 10% formalin. For fecal pellet collection, individual mice were placed in a sterile ventilated Plexiglas box (cleaned between animals) and fecal pellets were collected in sterile 1.5 mL Eppendorf tubes. The fecal pellets were immediately stored on dry ice prior to long-term storage at −80°C.

### Histopathology assessment

Mouse intestinal tissue from the ileum was fixed in 10% neutral buffered formalin. The formalin-fixed tissues were embedded in paraffin, cut into 5 μm sections, and stained with hematoxylin and eosin (H&E). The ileum histology slides were scanned using the Zeiss Axioscan.Z1. Following image export, villus height, villus width, crypt depth, and crypt counts were measured by a semi-blinded reviewer using four fields at 20X that were averaged for one animal using Zen 2 software (Supplementary Figure S1).

### Isolation of DNA and whole-genome sequencing

Total genomic DNA was extracted from the mouse fecal pellets using the FecalPower Pro Kit (Qiagen, Germantown, MD) according to manufacturer’s instructions without modifications. Briefly, a fecal pellet was added to lysis buffer in a PowerBead Pro tube and shaken on a vortex mixer and then centrifuged. Subsequently, inhibitors were removed, and DNA was bound to a spin column. DNA on the column was washed prior to elution. Concentrations were measured using the Qubit broad-range DNA kit as per the manufacturer’s instructions on a Qubit^®^ 4.0 Fluorometer (Life Technologies, Grand Island, NY). DNA was stored at −80°C prior to library preparation.

Libraries of DNA were constructed using the Illumina DNA PCR-Free workflow without modifications. Briefly, isolated DNA was mixed with bead-linked transposomes to tagment DNA at 41°C for 5 min. A stop buffer was added and then incubated at room temperature before being placed on a magnet stand and washed. Index adaptors were subsequently ligated with the sample mixture incubated at 37°C and 50°C for 5 min at each temperature. The resulting products were cleaned up with a final bead-based double size selection to ensure consistent fragment size of each sample. Library quantification was confirmed by quantitative PCR using the KAPA Library Quantification Kit-Illumina (Roche, Indianapolis, IN) before being pooled. Shotgun sequencing of libraries generated paired-end sequences using the Illumina NovaSeq 6000 platform (Illumina, San Diego, CA).

### Bioinformatic analysis

Raw Illumina paired-end sequencing data were processed using the Nextflow workflow nf-core/mag (github.com/nf-core/mag; v2.1.1) (27). Fastp and FastQC were used for quality control to remove adapter sequences and low-quality reads using the default parameter (28). Removal of PhiX and host DNA contamination was performed by aligning paired-end reads to the mouse reference genome (mm10) using Bowtie2 (29). Taxonomic assignment was performed using Kraken2 (v2.0.8) against the GTDB_release207_kraken2 database (30, 31). Following taxonomic classification, the remaining steps were conducted outside of the nf-core/mag pipeline. Kraken2 results were run through Bracken (v2.5.0) to acquire organism relative abundance values (32). Kraken-Biom was used to generate a Biom table, which was imported into Quantitative Insights Into Microbial Ecology (QIIME2, version 2021.8) (33, 34). Alpha and beta diversity analyses were conducted in QIIME2.

### Statistical analysis

Data analysis was performed in GraphPad Prism 10 (La Jolla, CA, United States). Values are shown as mean ± standard error of the mean (SEM). The difference among groups was analyzed by either two-way analysis of variance (ANOVA) with Dunnett’s or Tukey’s multiple comparisons for histology and alpha diversity measurements, respectively. Differences in beta diversity metrics were performed using the non-parametric permutational multivariate analysis of variance (PERMANOVA) and Adonis tests. The R (v 4.1.2) package qiime2R was used to create a phyloseq object from QIIME2. qza files and perform differential abundance evaluation with DESeq2 (35, 36). A *p*-value of <0.05 was considered significant.

## Results

### Effect of combination treatments on intestinal recovery following irradiation

**Figure 1:**
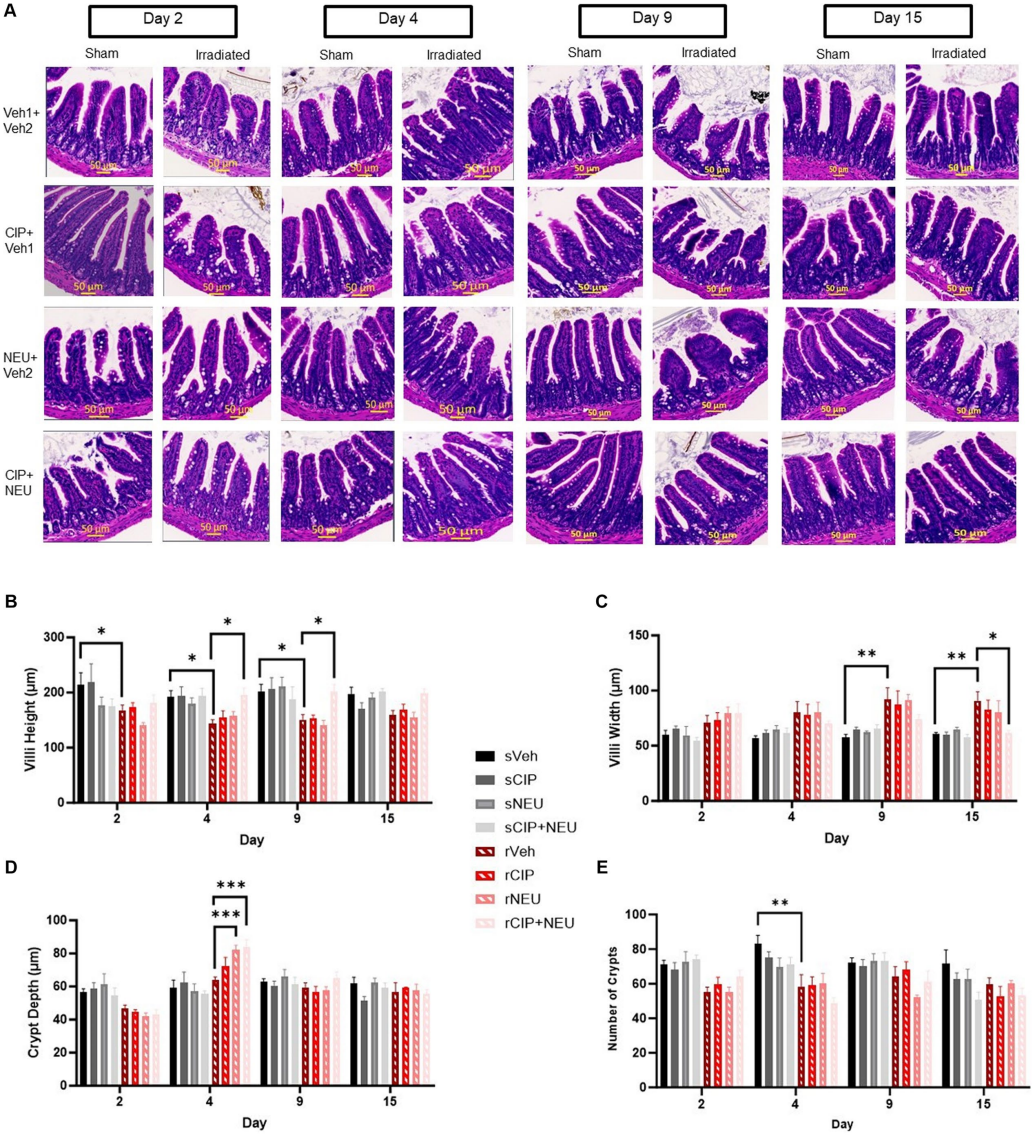
Effects of irradiation and treatments on intestinal histopathology. Panel **(A)** Representative H&E-stained images of cross sections of mouse ileum harvested on study days 2, 4, 9 or 15 in sham and irradiated animals with different treatments noted. Quantification of **(B)** villus height, **(C)** villus width, **(D)** crypt depth, and **(E)** crypt counts are depicted in sham, irradiated, and treatment groups. Data represent mean ± SEM (*n* = 6/group per time point). *p < 0.05; **p < 0.01; ***p < 0.001. Scale bar = 50 μm (yellow). The difference among groups was analyzed by two-way ANOVA.

Intestinal histopathology was evaluated to assess the extent of intestinal epithelial damage caused by radiation exposure and to monitor the efficacy of treatments. Representative images of H&E-stained ileum cross sections from each group are shown in Figure 1A. Radiation induced a reduction in villus height significant from days 2 to 9 (day 2, *p* = 0.028; day 4, *p* = 0.022; day 9, *p* = 0.012) and an increase in villus width starting at day 9 (*p* = 0.0096) continuing through 15 days (*p* = 0.033) compared to sham (Figures 1B,C). There were no changes in crypt depth from radiation; however, crypt counts were significantly reduced at day 4 post-irradiation (*p* = 0.0022) (Figures 1D,E). Treatment with NEU and/or CIP did not lead to significant differences in villus height or width at any time point post-irradiation. However, radiation-induced villus height reductions were significantly mitigated by CIP + NEU therapy at days 4 (*p* = 0.012) and 9 (*p* = 0.013) with villus blunting mitigated at day 15 (*p* = 0.04). At day 4 post-irradiation, crypt depth was significantly higher following NEU (*p* = 0.0001) and CIP + NEU (*p* = 0.0004) treatments. There were no significant differences in crypt counts observed in groups receiving therapy post-irradiation.

### Gut microbiota alpha and beta diversity analysis

**Figure 2:**
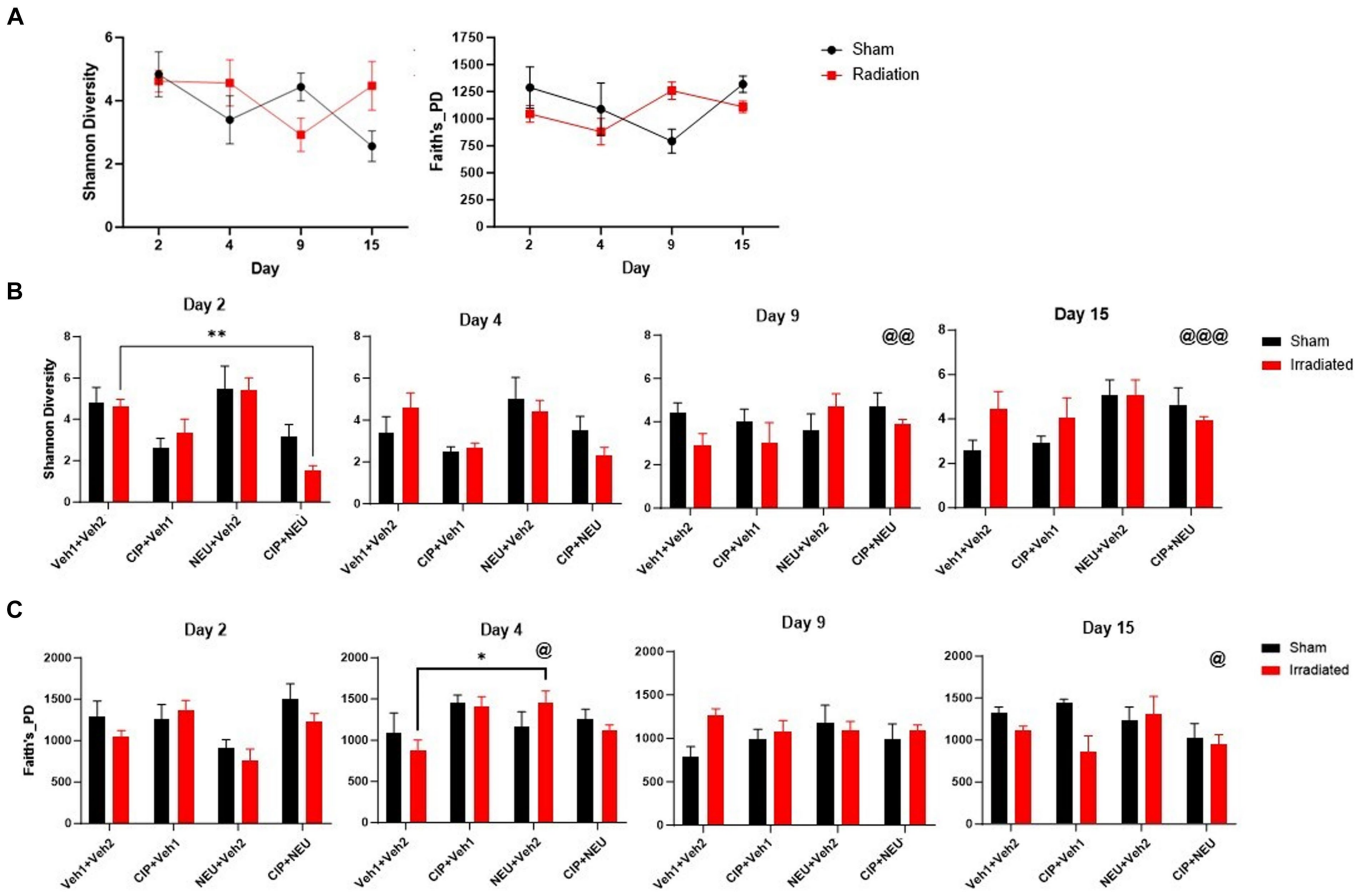
Impact of radiation exposure and treatments on taxonomic and phylogenetic alpha diversity metrics. **(A)** Shannon diversity and Faith’s phylogenetic diversity in sham and irradiated vehicle samples from mouse fecal pellets. **(B)** Longitudinal Shannon diversity in irradiated and treated animals following radiation. **(C)** Faith’s phylogenetic diversity at each time point in irradiated vehicle and treated mice post-irradiation. Alpha diversity values are represented as mean ± SEM. Statistical significance is indicated by text and symbol *p < 0.05; **p < 0.01. Day 2 (*n* = 6/group), day 4 (sVeh, *n* = 6; rVeh, *n* = 5; rCIP, *n* = 6; rNEU, *n* = 6; rCIP+PEG, *n* = 6), day 9 (sVeh, *n* = 6; rVeh, *n* = 5; rCIP, *n* = 6; rNEU, *n* = 6; rCIP+NEU, *n* = 5), and day 15 (sVeh, *n* = 6; rVeh, *n* = 4; rCIP, *n* = 5; rNEU, *n* = 4; rCIP+NEU, *n* = 6). Significant effect compared to day 2 denoted by text and symbol @ *p* < 0.05; @@ *p* < 0.01; @@@ *p* < 0.001. Alpha diversity was assessed with a two-way ANOVA.

To explore the relationship between radiation-induced and therapy-related histological changes and gut microbiota alterations, whole-genome sequencing of fecal samples was performed. Sequencing resulted in a mean of 2,561,412 reads per sample (range 1,239,099–5,899,196) after quality checks to include trimming and removal of host reads. Alpha diversity (within sample diversity) was measured by Shannon’s diversity and Faith’s phylogenetic diversity (Faith’s PD). Shannon diversity, which accounts for the abundance and evenness of organisms present, did not show significant differences between sham and irradiated animals irrespective of time point (Figure 2A). Similarly, radiation did not induce significant longitudinal differences in Faith’s PD, a phylogenetic measure that accounts for the amount of the phylogenetic tree covered by the microbial community. Treatment with either therapy alone did not significantly alter Shannon diversity, as NEU trended to increase (day 2, *p* = 0.91; day 4, *p* > 0.99; day 9, *p* = 0.43; day 15, *p* > 0.99), while CIP trended to decrease (day 2, *p* = 0.67; day 4, *p* = 0.37; day 9 and day 15, *p* > 0.99) (Figure 2B) Shannon diversity. However, CIP + NEU led to Shannon diversity decreases over the first two time points, significantly at day 2 (*p* = 0.0073), returning to non-treatment levels or higher by day 9 (rCIP+NEU day 2–9, *p* = 0.00073; day 2–15, *p* = 0.0007). Interestingly, Faith’s PD was non-significantly increased in the rCIP group early post-irradiation, decreasing over time (Figure 2C). NEU treatment induced a lower Faith’s PD post-irradiation at day 2 but generally increased over time, which was significantly higher on day 4 than day 2 (*p* = 0.041). While combined therapy non-significantly increased Faith’s PD compared to rVeh on days 2 and 4, a significant decrease was observed over time in this group (rCIP+NEU day 2–15, *p* = 0.037).

**Figure 3:**
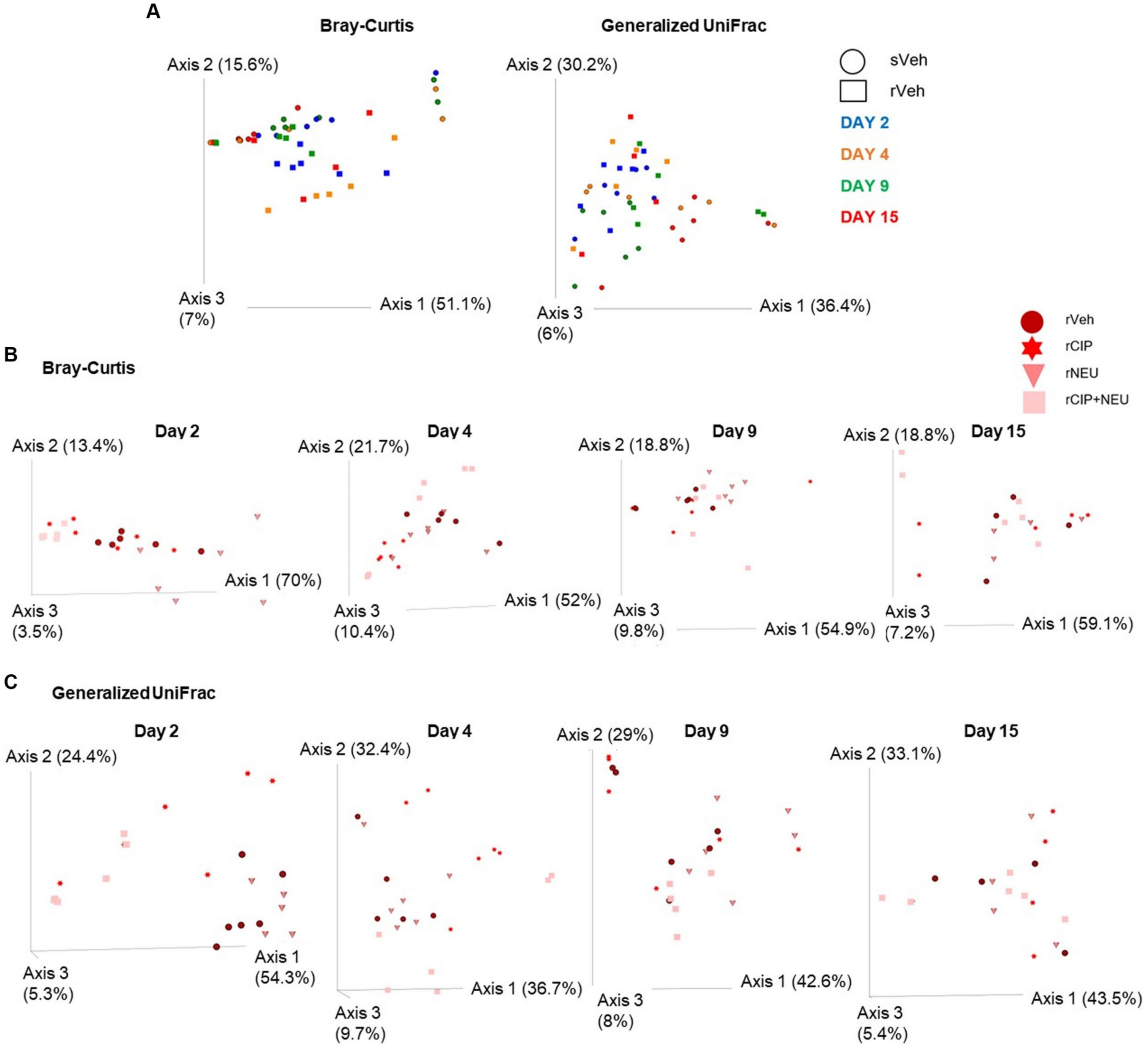
Beta diversity bacterial community clustering based on irradiation, treatment, and time. **(A)** Bray-Curtis, non-phylogenetic metric, and generalized UniFrac, phylogenetic measure, visualized by PCoA plots. Symbol shapes depict sham or irradiated samples, whereas colors represent different time points. **(B)** Represents Bray-Curtis PCoA plots of the effect of treatment following radiation at each time point (days 2, 4, 9, and 15), treatment type is denoted by different colors. **(C)** Generalized UniFrac shows colors representing different treatments following radiation at each study time point (days 2, 4, 9, and 15). Each data point represents a sample. The amount of variation explained by each axis are in parentheses. Day 2 (*n* = 6/group), day 4 (sVeh, *n* = 6; rVeh, *n* = 5; rCIP, *n* = 6; rNEU, *n* = 6; rCIP+NEU, *n* = 6), day 9 (sVeh, *n* = 6; rVeh, *n* = 5; rCIP, *n* = 6; rNEU, *n* = 6; rCIP+NEU, *n* = 5), and day 15 (sVeh, *n* = 6; rVeh, *n* = 4; rCIP, *n* = 5; rNEU, *n* = 4; rCIP+NEU, *n* = 6).

While alpha diversity refers to the number of distinct taxa and the evenness of these organisms identified within individual samples, beta diversity represents the disparities in microbial composition observed among different samples. Beta diversity was measured using Bray–Curtis (BC) and generalized UniFrac metrics to examine the effects of irradiation (Figure 3A) and treatment (Figures 3B,C). Bray–Curtis is a non-phylogenetic beta diversity measure with abundance included. Generalized UniFrac accounts for phylogeny diversity and controls for the weight put on abundant lineages. Bray–Curtis revealed that the inclusion of microbial abundance led to a clear separation between sham and irradiated groups, which was significant at days 2 (*p* = 0.023) and 15 (*p* = 0.009). We observed similar trends when evaluating generalized UniFrac, which revealed a significant difference due to irradiation at days 2 (*p* = 0.045), 9 (*p* = 0.002), and 15 (*p* = 0.013) (Figure 3A; Supplementary Table S1). Treatment with CIP led to significant community differences compared to rVeh only on day 4 (*p* = 0.007) with no significant shifts from sVeh across time (Figure 3B). NEU-treated samples clustered significantly different than both vehicle-treated cohorts initially at day 2 (rNEU-sVeh, *p* = 0.011; rNEU-rVeh, *p* = 0.017) and compared to rVeh at day 9 (*p* = 0.03). CIP + NEU also led to significant community shifts at days 2 (*p* = 0.002), 4 (*p* = 0.043), and 15 (*p* = 0.002) compared to sVeh, but only at day 2 when compared to rVeh (*p* = 0.005). Accounting for phylogenetic relationships revealed that CIP and CIP + NEU shifted beta diversity from sVeh and rVeh more significantly than NEU treatment over the course of the study (Figure 3C).

### Taxonomic profiling of gut microbiota

**Figure 4:**
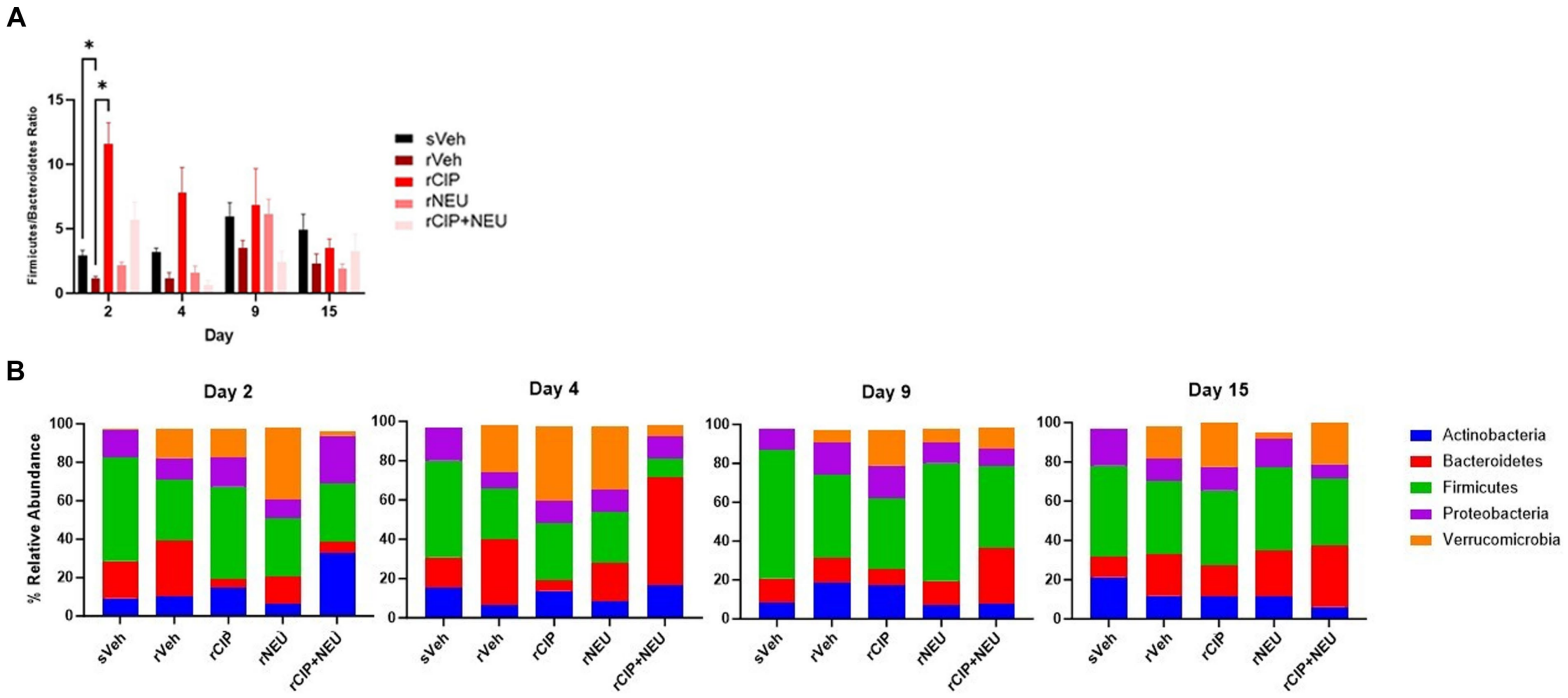
Bacterial phyla level taxonomic classifications. **(A)** Comparison of Firmicutes/Bacteroidetes (F/B) ratio among sham and irradiated groups with or without treatment. Bars represent the mean relative abundance of Firmicutes and Bacteroidetes phyla measured in fecal pellets of animals collected on days 2, 4, 9, and 15. Data represent mean ± SEM. Significant differences are indicated in the text and symbol *p < 0.05. **(B)** Phyla in sham and irradiated vehicle samples compared to treatment following radiation exposure at each time point (days 2, 4, 9, and 15). Stacked bar plots show the mean relative abundance. Phyla consisting of ≥ 1% of total bacterial composition. Day 2 (*n* = 6/group), day 4 (sVeh, *n* = 6; rVeh, *n* = 5; rCIP, *n* = 6; *r*NEU, *n* = 6; rCIP+NEU, *n* = 6), day 9 (sVeh, *n* = 6; rVeh, *n* = 5; rCIP, *n* = 6; rNEU, *n* = 6; rCIP+NEU, *n* = 5), and day 15 (sVeh, *n* = 6; rVeh, *n* = 4; rCIP, *n* = 5; rPEG, *n* = 4; rPEG+CIP, *n* = 6).

**Figure 5:**
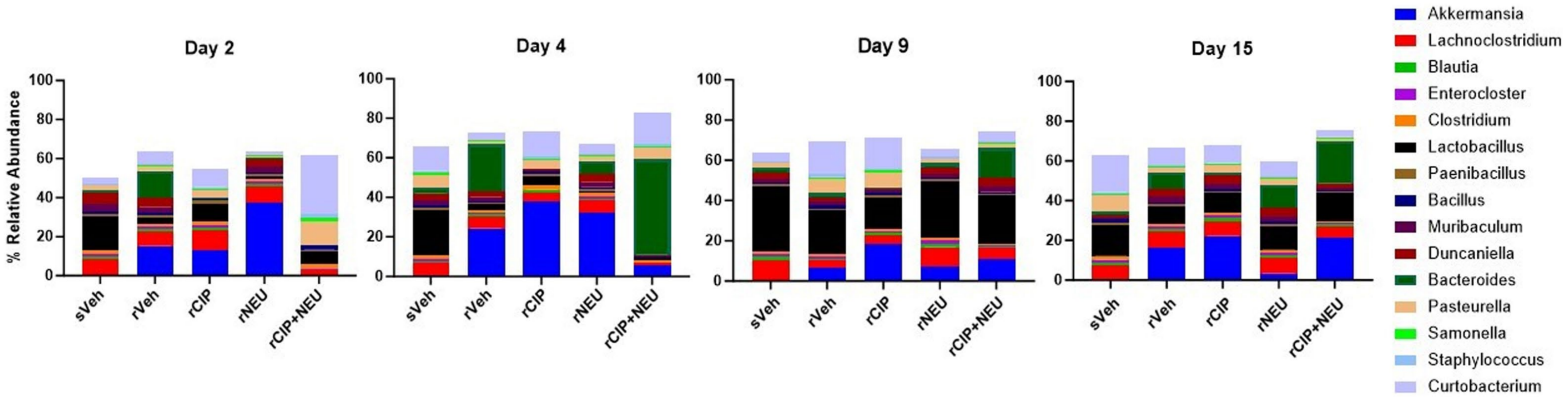
Genus-level taxonomy at days 2, 4, 9, and 15 showing the effect of radiation and treatment following irradiation. Barplots represent the mean relative abundance of the genera >1%. Day 2 (*n* = 6/group), day 4 (sVeh, *n* = 6; rVeh, *n* = 5; rCIP, *n* = 6; rNEU, *n* = 6; rCIP+NEU, *n* = 6), day 9 (sVeh, *n* = 6; rVeh, *n* = 5; rCIP, *n* = 6; rNEU, *n* = 6; rCIP+NEU, *n* = 5), and day 15 (sVeh, *n* = 6; rVeh, *n* = 4; rCIP, *n* = 5; rNEU, *n* = 4; rCIP+NEU, *n* = 6).

Next, we examined taxonomic alterations due to irradiation and treatment and started with the Firmicutes/Bacteroidetes (F/B) ratio as an established measure of microbial stability (37). Irradiation consistently lowered the F/B compared to sham, which was significant at day 2 (*p* = 0.047) (Figure 4A). The lone significant difference in F/B after treatment following radiation was in the rCIP group at day 2 (*p* = 0.014), which increased compared to rVeh. Phyla differences post-irradiation were most apparent in Firmicutes, Bacteroidetes, and Verrucomicrobia (Figure 4B; Supplementary Figure S2). Irradiation decreased the relative abundance of Firmicutes compared to sham, which was significant at day 4 (log_2_ fold change [FC] = −2.23, *p* = 0.003) and 9 (FC = −2.59, *p* = 0.00043). Bacteroidetes slightly increased following irradiation but was not significant at any time point (day 4, *p* = 0.56). Interestingly, irradiation greatly increased the abundance of Verrucomicrobia, with the greatest difference from sVeh at day 15 (FC = 6.92, *p* = 0.000011). At day 2 post-irradiation, CIP significantly decreased Bacteroidetes (FC = −1.54, *p* = 0.017), which continued through day 4 (FC = −2.50, *p* = 0.0028). NEU treatment led to increased Verrucomicrobia at days 2 and 4 compared to rVeh with similar profiles to sVeh at days 9–15. Interestingly, at day 2 post-irradiation, CIP + NEU led to the highest prevalence of Actinobacteria (32.79 ± 1.65%) and Proteobacteria (25.01 ± 3.01%) of any group or time point, and on day 4, Bacteroidetes (55.26 ± 14.51%) had the highest abundance in the study. At day 2, rCIP+NEU induced decreases in Verrucomicrobia (FC = −4.89, *p* = 0.000071) and Bacteroidetes (FC = −2.81, *p* = 0.00081) that were significant compared to rVeh. Evaluation of genus-level taxonomy revealed that irradiation resulted in longitudinal alterations in *Lactobacillus*, *Bacteroides*, *Akkermansia, Curtobacterium*, and *Pasteurella* compared to sham animals (Figure 5; Supplementary Figure S3). At each time point, *Akkermansia* and *Bacteroides* were both increased post-irradiation (*Akkermansia*, day 2—FC = 5.06, *p* = 0.001; day 4—FC = 4.00, *p* = 0.0078; day 9—FC = 5.48, *p* = 0.0001; day 15—FC = 14.18, *p* = 3.8×10^−17^; *Bacteroides*, day 2—FC = 6.62, *p* = 5.6×10^−10^; day 4—FC = 1.63, *p* = 0.098; day 15—FC = 0.55, *p* = 0.19). *Bacteroides thetaiotaomicron* and *Akkermansia muciniphila* were the predominating species in each genus that showed significant alterations. Although not significant, *Lactobacillus* decreased post-irradiation at each time point. CIP treatment mitigated the loss of *Lactobacillus* at days 2–4 more than other treatments and significantly decreased *Bacteroides* post-irradiation (day 2—FC = −6.52, *p* = 7.5×10^−10^; day 4—FC = −7.00, *p* = 3.2×10^−11^; day 4—FC = −1.56, *p* = 0.043; day 15—FC = −5.19, *p* = 0.00093). *Lactobacillus johnsonii* was the most prevalent *Lactobacillus* species driving alterations. At days 2 and 4, NEU treatment exacerbated the radiation-induced increase in *Akkermansia* prevalence while *Lactobacillus* and *Bacteroides* (day 2 – FC = −6.36, *p* = 5.3×10^−9^) abundance was decreased. These effects of NEU were transient, however, with no significant differences at days 9 or 15. Similarly, early on CIP + NEU led to unique genus-level composition compared to other groups as on day 2, rCIP+PEG increased *Curtobacterium* (FC = 2.08, *p* = 0.00001) and *Pasteurella* (FC = 2.25, *p* = 0.00001) while on day 4 there was a shift to *Bacteroides* (day 2–4: FC = 9.12, *p* = 5.31×10^−19^). On days 9 and 15, rCIP+NEU had a higher abundance of *Akkermansia* and *Bacteroides* (FC = 3.57, *p* = 0.006) than rVeh; however, *Lactobacillus* levels were not different.

## Discussion

The interplay between H-ARS and GI-ARS may have significant implications for host response and survival following radiation exposure (17, 38, 39). Gut microbiota alterations have been shown post-irradiation, as certain commensal flora are linked to positive responses and outcomes reducing radiotoxicity (11, 40–42). However, there is limited knowledge on the longitudinal effects of high-dose radiation injury and FDA-approved medical countermeasures on the gut microbiota. To this end, we performed shotgun sequencing on DNA isolated from mouse fecal pellets to evaluate alterations in the gut microbiota following irradiation and treatment with Neulasta and/or ciprofloxacin. We examined GI-ARS histologically and showed that radiation induced a reduction in villus height and crypt counts and an increase in villus width. CIP + NEU therapy mitigated villus height and width alterations over time. We did not observe a strong relationship between alpha diversity and phyla relative abundance on histological data with none of the linear regression analyses revealing a significant relationship (data not shown). Analysis of beta diversity and taxonomic classifications of gut flora post-irradiation provided more insight than alpha diversity metrics into longitudinal alterations. CIP treatment had measurable negative consequences on Shannon diversity with inhibition of Bacteroidetes and increased Verrucomicrobia. While NEU increased Shannon diversity and Firmicutes abundance over time with similar profiles to sVeh at the study endpoint. CIP + NEU induced a decrease in Shannon diversity and unique taxonomic profiles at the first two time points, returning closer to non-treatment levels over time. Co-therapy also led to significant beta diversity shifts post-irradiation from sham than NEU or CIP alone.

To our knowledge, this study is the first to use shotgun sequencing to evaluate gut microbiota in a pre-clinical animal model following total body irradiation. We did not observe any significant differences in alpha diversity between sham and irradiated gut microbiota throughout the 15-day study period. While large animal studies evaluating alpha diversity post-irradiation also show little changes over time, these studies are typically shorter in duration (3–4 days) and focus on altered relative abundance of certain species (19, 20, 22). Alternatively, rodent models show irradiation consistently lowers the often used Shannon diversity metric, which we also show until day 9 (23, 42–47). Although alpha diversity analysis did not lead to significant differences, we detected significant shifts in beta diversity post-irradiation throughout the study period consistent with both previous rodent and large animal models (22, 44, 46).

The F/B ratios were decreased post-irradiation, which was driven by increases in Bacteroidetes and decreases in Firmicutes, which aligns with previous reports (22, 23, 45, 46). Most studies evaluating gut microbiota post-irradiation have also found that decreased Firmicutes strongly contribute to the altered F/B ratio (23). However, the prevalence of Bacteroides, the second most prevalent intestinal phyla, differs from non-human primate (NHP) models that show increases in Bacteroidetes, specifically the *Bacteroides* (19, 22). However, results from other rodent models and a mini pig model describe a decrease in Bacteroidetes abundance (20, 44, 46, 48). We found that the commensal organism *Bacteroides thetaiotaomicron* drove the *Bacteroides* increase following radiation consistently accounting for over 90% of the increase (data not shown). Interestingly, this bacterium produces several short-chain fatty acids (SCFAs) and has been shown to modulate immune function contributing to gut homeostasis (41). Other previously reported irradiation-induced phyla changes are increased Proteobacteria and Verrucomicrobia (19, 20, 43, 44, 48–50). While we saw significant increases in Verrucomicrobia abundance following radiation, no major shifts in Proteobacteria were observed.

This increase in Verrucomicrobia was due to the common gut flora, *Akkermansia muciniphila*, which contributes to the degradation of mucin, production of SCFAs, and improvements in glucose metabolism (40). This bacterium is commercially available as a probiotic, but its increase in abundance in trauma patients who have died suggests a potential negative effect (51). Similarly, there are mixed reports on the role that *A. muciniphila* plays following radiation (23, 42, 50, 52, 53). Tian et al. (52) used an oral gavage of *Akkermansia* to demonstrate increases in mouse survival over a 30-day period following irradiation with enhanced production of SCFAs. Conversely, *Akkermansia* has been associated with inflammation as it is consistently found as an organism more abundant following irradiation and in colitis models (42, 50, 52–54). Gerassy-Vainberg et al. (50) demonstrated that radiation induces a proinflammatory state with increased *Akkermansia*. Other studies examining the protective role versus a potential detrimental role of this particular bacteria post-irradiation are warranted.

Although other genus-level differences are varied across studies, *Alistipes* was consistently reported to be more abundant post-irradiation, whereas *Lactobacillus* was significantly reduced (23). It has been well established that *Lactobacillus* is a beneficial commensal genus well touted as a probiotic candidate in many GI diseases and shown to aid in recovery from radiation-related injury (23, 45, 55, 56). We did not observe a high abundance of *Alistipes* or *Prevotella* within the gut microbiota, which have also been described as potential therapeutic targets post-irradiation. The effects of P*revotella* are not well understood as some data suggest *Prevotella* is associated with survival following radiation (19, 20, 22), while other literature has linked *Prevotella* spp. with gut inflammation (57). Similarly, the role these bacteria play in post-irradiation changes and treatment warrants future examination. Moreover, while CIP mitigated some of the irradiation-induced changes (e.g., lower *Lactobacillus*) co-administration with other probiotics may prove beneficial for GI-ARS.

While assessing the effects of ionizing radiation *per se* on the gut microbiota is critical in identifying alterations and potential targets, response to a mass casualty nuclear/radiological event would be coupled with supportive care to include pharmacotherapies. To this end, the evaluation of gut microbiota following the administration of FDA-approved radiation mitigators is of great interest. Li et al. (58) used 9.25 Gy of total body irradiation in mice to examine the effects of G-CSF and an anti-apoptotic agent on the gut microbiota and animal survival over 30 days. Although the authors noted that there was limited evidence that G-CSF had a direct effect on the microbiota, results showed *Lactobacillus* and *Ruminococcus* were predictors of increased survival, and *Akkermansia* negatively correlated with survival (58). Alpha diversity generally increased following NEU administration post-irradiation, which seemed to be driven more by species richness than phylogenetic diversity. Herein, NEU led to microbial communities that clustered closer and had more similar taxonomic profiles to sham than CIP or co-therapy post-irradiation. It is noteworthy that we used a higher dose, as well as repeated doses of NEU, and analyzed it with concomitant antibiotic usage. Unfortunately, we were unable to address whether the microbiome changes of NEU itself were associated with any effect on apoptosis or cell turnover. Ciprofloxacin is a broad-spectrum antibiotic in the fluoroquinolone class that has demonstrated efficacy against *Enterobacteriaceae* and has an impact on the gut microbiota (59). Interestingly, CIP + NEU treatment appeared to mirror the impact of CIP in terms of alpha diversity findings, suggesting a larger impact of CIP compared to NEU. Beta diversity and taxonomic alterations seemed to involve an interaction between NEU and CIP that led to distinct microbial communities and composition, which did start normalizing by day 9. This highlights the polymicrobial nature of the gut and also suggests that multiple treatments may result in repeated perturbations on the gut microbiome.

GI-ARS usually follows radiation exposure at a higher dose than H-ARS; thus, there could be an interplay of myelosuppression and GI injury. There is adequate evidence on the efficacy of FDA-approved growth factors mitigating the effects of H-ARS and improving survival in a variety of animal models (12, 15, 17, 38, 39). Recently, the effect of these FDA-approved treatments on the incidence and severity of GI-ARS was investigated using this mouse model consisting of 9.5 Gy ^60^Co γ-photon total body radiation. Kiang et al. (17) examined the effect of the four FDA-approved drugs on GI injury showing minimal efficacy while Neulasta coupled with ciprofloxacin was significantly better in the improvement of GI injury and brain hemorrhage. Evidence suggests that gut microbiota play an important role in maintaining intestinal homeostasis in health, injury, and disease (7). Furthermore, gut microbiota modulation has been shown to induce an expansion of bone marrow granulocyte monocyte precursors, gut neutrophil infiltration, and protection from pathogen colonization (60). Thus, identifying alterations and contributions of specific microbiota post-irradiation are important for elucidating their role in host homeostasis and reveal more about the interplay among these organ systems. To gain further mechanistic insight into how bacterial alterations post-irradiation impact host response, germ-free animals with single or mixed bacterial isolates should be tested.

There are some limitations of this study worth mentioning. Mouse models are not ideal for the evaluation of the gut microbiome (61), although ethical barriers to studying the effect of high-dose total body irradiation in humans necessitate animal studies as highlighted by the Animal Rule. However, value remains in the evaluation of gut microbiota in mouse models post-injury and in various disease states considering evidence has been extrapolated to humans (62). Additionally, the approach and model presented herein have many findings in agreement with previous NHP models, which are the gold standard animal for studying ARS due to the similarities in physiological responses and organ structure to humans (19, 22). Other limitations include the use of exclusively female mice, our sample size per group (*n* = 6) which may have underpowered some clinically meaningful changes, and the lack of circulating cytokine or blood cell biomarker data. However, Kiang et al. (17) observed following radiation exposure, CIP + NEU helped recover platelet counts and inhibit serum IL-18 increases (17). In addition, we focused on bacteria changes given their dominance in the gut but there could be value in examining, for example, mycobiome changes. Moreover, although we used high-dose total body irradiation that should induce GI-ARS, our study was designed to address specific microbial alterations post-irradiation and treatment. As such, we were not able to provide the relationship of microbial changes with GI symptoms, animal survival, or other key intestinal endpoints. Finally, no baseline data were available to compare changes in individual subjects, and thus, we relied on sham and vehicle-treated animals for comparisons.

Despite these limitations, our study adds to the body of literature on the effect of high-dose total body radiation on the gut microbiota and is the first report demonstrating the effect of two supportive therapies, Neulasta and ciprofloxacin, on the gut microbiota following irradiation. The impact of supportive care and therapeutics post-irradiation is an important consideration as iatrogenic influences affect the microbiome. As bacteria represent a completely modifiable factor, additional exploration into microbial changes post-irradiation and treatment can provide strategies for prophylactic or reactive interventions for maintenance or restoration of gut commensal homeostasis to improve overall survival and clinical outcomes. Specifically, accumulating evidence suggests that further mechanistic study of *Akkermansia*, *Bacteroides*, *Lactobacillus*, and *Prevotella* is warranted. Future studies should identify whether increased prevalence of *Akkermansia* and *Bacteroides* post-irradiation is detrimental or whether a host defense response required for SCFA production in order to return to gut homeostasis. This study used an unbiased sequencing approach to provide critical knowledge of radiation-induced gut microbiome changes and alterations subsequently involving pharmacotherapy to uncover targets for supplementation and potential therapeutics.

## Data availability statement

The data presented in this study are publicly available and can be found here: https://www.ncbi.nlm.nih.gov/bioproject/1086397, PRJNA1086397.

## Ethics statement

The animal study was approved by Uniformed Services University of the Health Sciences. The study was conducted in accordance with the local legislation and institutional requirements.

## Author contributions

TH: Writing – review & editing, Writing – original draft, Visualization, Methodology, Investigation, Formal analysis, Data curation. AF: Formal analysis, Methodology, Software, Visualization, Writing – original draft, Writing – review & editing. GC: Data curation, Investigation, Methodology, Writing – original draft, Writing – review & editing. MZ: Writing – review & editing, Writing – original draft, Investigation, Data curation. MO: Writing – review & editing, Writing – original draft, Methodology, Investigation, Data curation. BL: Data curation, Investigation, Writing – original draft, Writing – review & editing. XL: Writing – review & editing, Writing – original draft, Methodology, Investigation, Data curation. MX: Conceptualization, Data curation, Investigation, Methodology, Project administration, Writing – review & editing, Writing – original draft, Supervision. JK: Conceptualization, Data curation, Formal analysis, Funding acquisition, Investigation, Methodology, Project administration, Resources, Supervision, Writing – original draft, Writing – review & editing. DB: Formal analysis, Funding acquisition, Project administration, Resources, Supervision, Visualization, Writing – original draft, Writing – review & editing. LH: Data curation, Investigation, Writing – review & editing.

## Supplementary material

Supplementary Figure 1 Mouse small intestine cross section representative H&E-stained image with yellow labeled lines to depict how measurement of villus height, villus width, and crypt depth were performed. Asterisks represent crypt cell counts which are not fully inclusive only representative.

SUPPLEMENTARY FIGURE 2 Normalized counts from DESeq2 differential abundance analysis reveal key phyla. **(A)** Colored boxplots represent sham or irradiated samples at each timepoint (days 2, 4, 9, and 15) for Bacteroidetes, Firmicutes, Proteobacteria, and Verrucomicrobia. **(B)** Schematic illustrating each treatment following radiation compared to radiation and vehicle over time and phyla.

SUPPLEMENTARY FIGURE 3 DESeq2 differential abundance normalized counts for selected genera to include Akkermansia, Bacteroides, and Lactobacillus at each study time point and treatment represented by different colors compared to radiation and vehicle. **(A)** Represents genus level count differences based on injury pattern. **(B)** Normalized counts comparing treatments following radiation.

Supplementary Table 1 Permutational multivariate analysis of variance (PERMANOVA) table of different beta diversity metrics showing generated p-values and significant effects of radiation and treatment on gut microbiota pairwise comparisons at each time point.
