# Supplementary figures and images for: Effects of combined ciprofloxacin and Neulasta therapy on intestinal pathology and gut microbiota after high-dose irradiation in mice
Source: Front Public Health. 2024 May 14;12:1365161. doi: 10.3389/fpubh.2024.1365161 (PMC11130442; doi:10.3389/fpubh.2024.1365161)

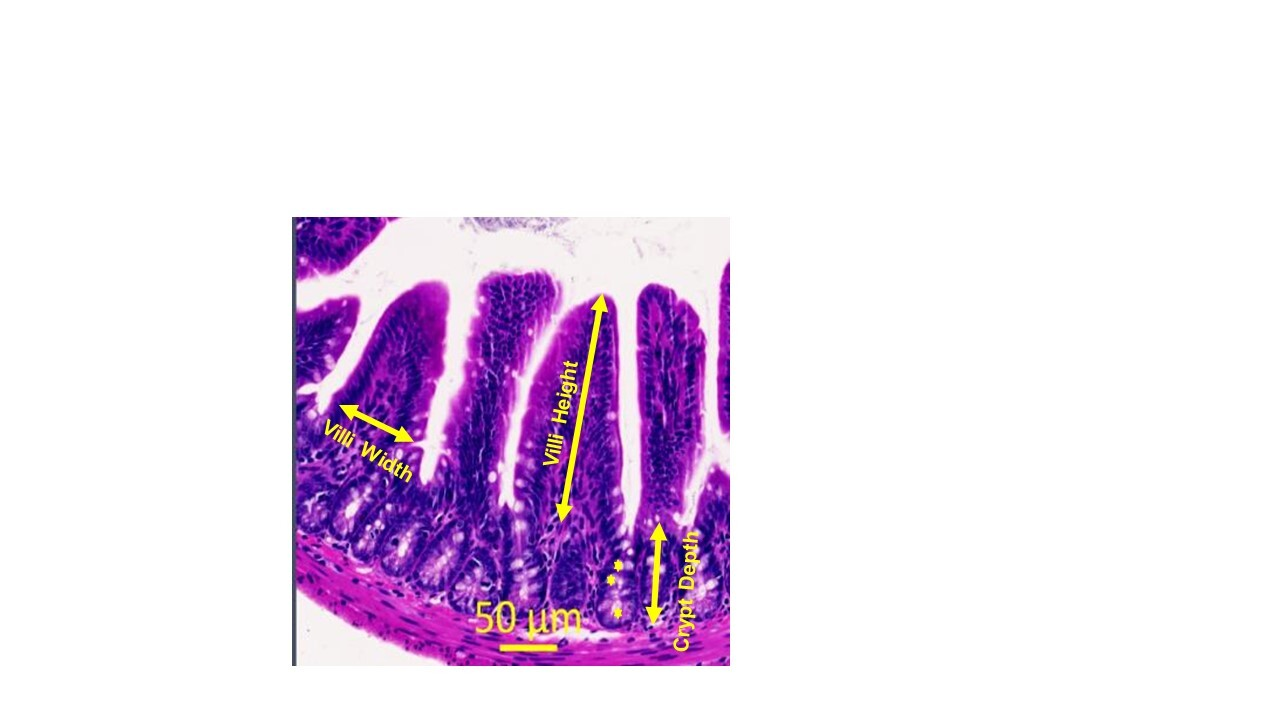

Supplement: Supplementary Figure 1 — Mouse small intestine cross section representative H&E-stained image with yellow labeled lines to depict how measurement of villus height, villus width, and crypt depth were performed. Asterisks represent crypt cell counts which are not fully inclusive only representative. [file Image_1.tif]

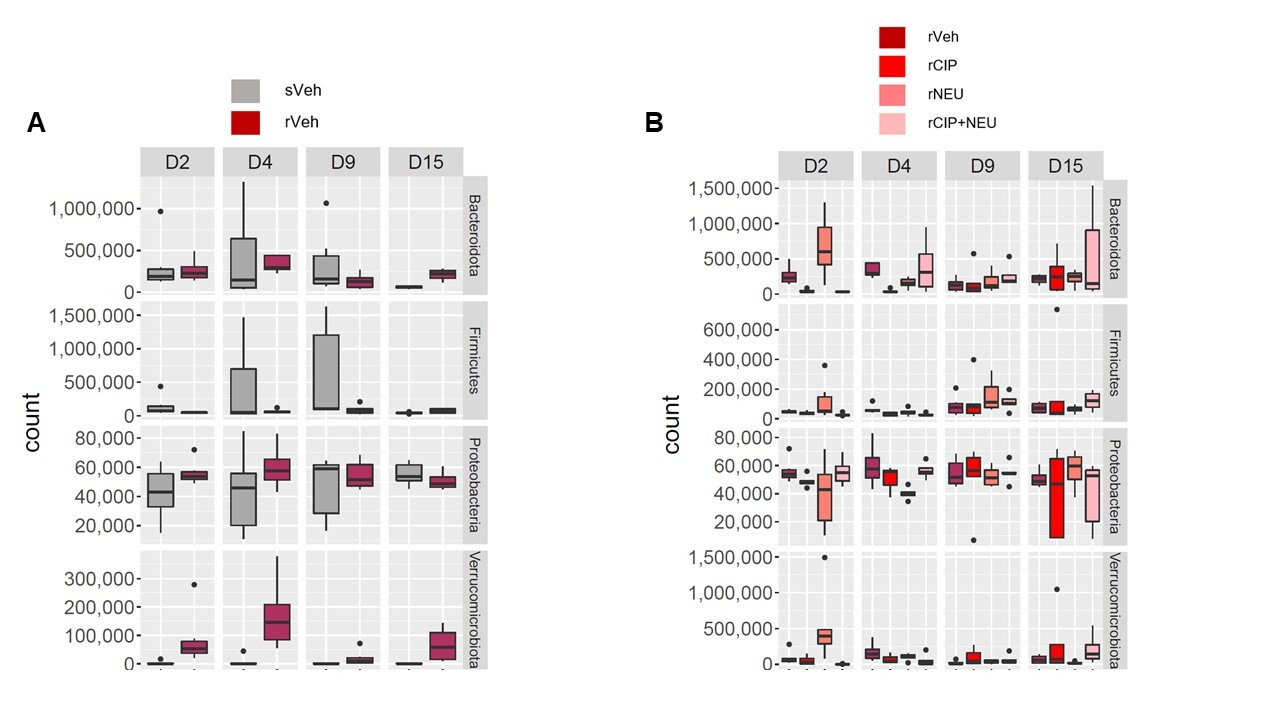

Supplement: SUPPLEMENTARY FIGURE 2 — Normalized counts from DESeq2 differential abundance analysis reveal key phyla. (A) Colored boxplots represent sham or irradiated samples at each timepoint (days 2, 4, 9, and 15) for Bacteroidetes, Firmicutes, Proteobacteria, and Verrucomicrobia. (B) Schematic illustrating each treatment following radiation compared to radiation and vehicle over time and phyla. [file Image_2.tif]

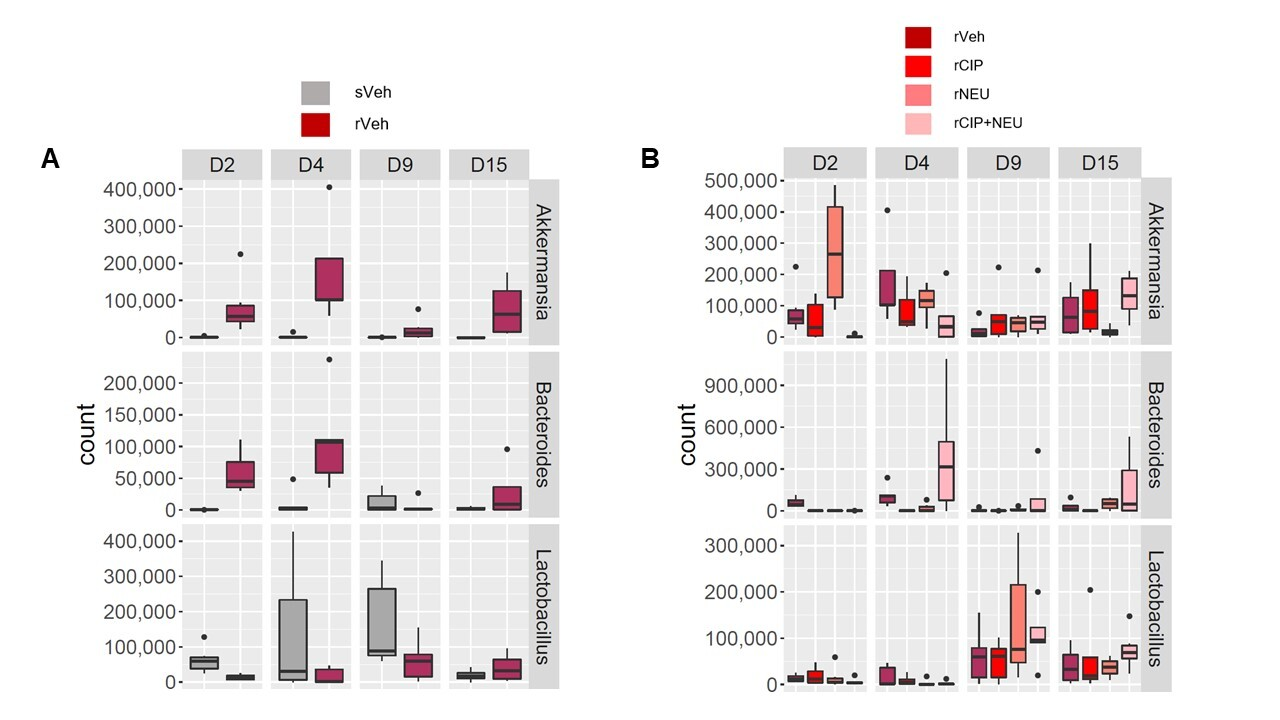

Supplement: SUPPLEMENTARY FIGURE 3 — DESeq2 differential abundance normalized counts for selected genera to include Akkermansia, Bacteroides, and Lactobacillus at each study time point and treatment represented by different colors compared to radiation and vehicle. (A) Represents genus level count differences based on injury pattern. (B) Normalized counts comparing treatments following radiation. [file Image_3.tif]
