# Supplementary material for: Effects of combined ciprofloxacin and Neulasta therapy on intestinal pathology and gut microbiota after high-dose irradiation in mice
Source: Front Public Health. 2024 May 14;12:1365161. doi: 10.3389/fpubh.2024.1365161 (PMC11130442; doi:10.3389/fpubh.2024.1365161)
Supplement: Supplementary Table 1 — Permutational multivariate analysis of variance (PERMANOVA) table of different beta diversity metrics showing generated p-values and significant effects of radiation and treatment on gut microbiota pairwise comparisons at each time point. [file Table_1.DOCX]

| **Metric** | **Comparison (p-value)** | **Day 2** | **Day 4** | **Day 9** | **Day 15** |
| --- | --- | --- | --- | --- | --- |
| **Bray-Curtis** | sham v radiation sham v rCIP sham v rNEU sham v rCIP+NEU rad v rCIP rad v rNEU rad v rCIP+NEU | 0.023 0.072 0.011 0.002 0.066 0.017 0.005 | 0.18 0.08 0.15 0.043 0.007 0.60 0.078 | 0.073 0.15 0.49 0.19 0.72 0.03 0.074 | 0.009 0.081 0.081 0.002 0.70 0.83 0.36 |
| **Jaccard** | sham v radiation sham v rCIP sham v rNEU sham v rCIP+NEU rad v rCIP rad v rNEU rad v rCIP+NEU | 0.22 0.10 0.49 0.004 0.069 0.093 0.002 | 0.73 0.059 0.17 0.12 0.038 0.32 0.068 | 0.094 0.12 0.50 0.23 0.39 0.048 0.15 | 0.11 0.33 0.065 0.017 0.53 0.92 0.54 |
| **UW Unifrac** | sham v radiation sham v rCIP sham v rNEU sham v rCIP+NEU rad v rCIP rad v rNEU rad v rCIP+NEU | 0.13 0.18 0.045 0.009 0.018 0.039 0.004 | 0.37 0.062 0.26 0.21 0.003 0.026 0.016 | 0.012 0.23 0.20 0.12 0.65 0.12 0.08 | 0.012 0.006 0.13 0.006 0.29 0.57 0.33 |
| **W Unifrac** | sham v radiation sham v rCIP sham v rNEU sham v rCIP+NEU rad v rCIP rad v rNEU rad v rCIP+NEU | 0.062 0.002 0.003 0.008 0.004 0.065 0.003 | 0.13 0.042 0.10 0.068 0.01 0.69 0.03 | 0.002 0.042 0.44 0.002 0.71 0.03 0.037 | 0.012 0.082 0.095 0.003 0.58 0.88 0.55 |
| **Gen Unifrac** | sham v radiation sham v rCIP sham v rNEU sham v rCIP+NEU rad v rCIP rad v rNEU rad v rCIP+NEU | 0.045 0.003 0.006 0.002 0.004 0.073 0.005 | 0.13 0.034 0.068 0.065 0.005 0.71 0.022 | 0.002 0.043 0.45 0.004 0.76 0.033 0.030 | 0.013 0.060 0.10 0.002 0.61 0.90 0.54 |
